# Case report: Durable response after pembrolizumab in combination with radiation - induced abscopal effect in platinum - refractory metastatic endometrial clear cell carcinoma

**DOI:** 10.3389/fimmu.2022.1079253

**Published:** 2022-12-15

**Authors:** Chien-Hsiang Kao, Chien-Ting Liu, Hao Lin, Yung-Cheng Huang, Jui Lan, Yu-Che Ou, Hung-Chun Fu, Chen-Hsuan Wu

**Affiliations:** ^1^ Department of Obstetrics and Gynecology, Kaohsiung Chang Gung Memorial Hospital and Chang Gung University College of Medicine, Kaohsiung, Taiwan; ^2^ Division of Hematology-Oncology, Department of Internal Medicine, Kaohsiung Chang Gung Memorial Hospital, Chang Gung University, College of Medicine, Kaohsiung, Taiwan; ^3^ Department of Nuclear Medicine, Kaohsiung Chang Gung Memorial Hospital, Chang Gung University, College of Medicine, Kaohsiung, Taiwan; ^4^ Department of Anatomic Pathology, Kaohsiung Chang Gung Memorial Hospital and Chang Gung University College of Medicine, Kaohsiung, Taiwan; ^5^ Department of Obstetrics and Gynecology, Chia-Yi Chang Gung Memorial Hospital, Chia-Yi, Taiwan; ^6^ Graduate Institute of Clinical Medical Sciences, College of Medicine, Chang Gung University, Taoyuan, Taiwan

**Keywords:** endometrial cancer, abscopal effect, pembrolizumab, immunotherapy, clear cell carcinoma, ARID1A, radiotherapy, PD-L1

## Abstract

Advanced endometrial clear cell carcinoma (CCC) tends to have poor prognosis owing to aggressive clinical behavior and poor response to conventional chemotherapy. Herein, we report a case of platinum-refractory recurrent ECCC successfully treated with the combination of pembrolizumab, localized radiotherapy and a few cycles of chemotherapy with an extremely durable response even after cessation of immunotherapy for 3 years at the time of publication.

## 1 Introduction

Endometrial carcinoma (EC) is the most common gynecologic malignancy, with an estimated incidence rate of 15.6-19.1 per 100,000 in North America and Western Europe (1). In Taiwan, uterine corpus carcinoma was reported to be the seventh most common type of cancer in women in an annual cancer registry report in 2017 (2). Endometrial clear cell carcinoma (ECCC) accounts for only 1% to 5.5% of all uterine carcinomas, and it tends to present clinically as an aggressive ‘Type II’ pathogenetic type (3, 4) compared to the endometrioid subtype of EC. In addition, it tends to develop chemoresistance, which contributes to a poorer prognosis (5, 6) with a 5-year overall survival (OS) rate of less than 40% in patients with advanced stage disease. Currently, conventional systemic therapy for metastatic or recurrent EC mainly consists of combination platinum-based chemotherapy and either taxane or epirubicin. However, standard treatment algorithms after failure of first-line systemic treatment or repeated recurrence have a poor response rate. Along with new molecular classification of EC proposed by The Cancer Genome Atlas (TCGA) (7), two subgroups of POLE and MSI-high harboring higher tumor mutation burden are expected to have better response to immunotherapy. FDA further approved Pembrolizumab use in 2017 for MSI-high or dMMR solid tumors (8). The concept of risk stratification based on molecular signatures enlighten the importance of specific therapeutic strategies including immune checkpoint inhibitor and other novel targeted therapies as well as exploration of possible prognostic markers, included the status of genes such as PTEN, ARIDIA, PIK3CA, PIK3RI, and KRAS for EC (9).

## 2 Case description

**Figure 1:**
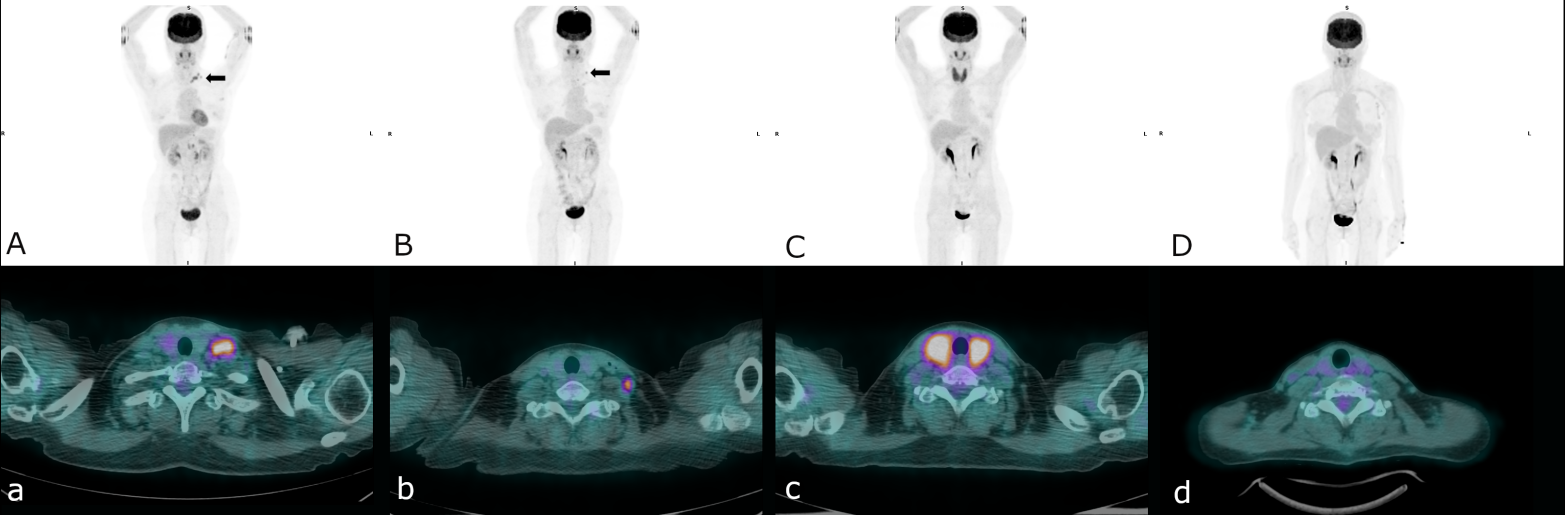
A serial pelvic MRI: T2-weighted sagittal and DWI-axial views and PET/CT images during treatment and follow-up **(A)** First recurrence at left supraclavicular lymph nodes (arrow), **(B)** Second recurrence at left lower neck lymph nodes (arrow), **(C)** Complete remission of second recurrence, thyroiditis was also noted, **(D)** Sustained free of disease since stopping immunotherapy.

A 52-year-old female, gravida 2, para 2, with no remarkable family gynecological or colorectal cancer history presented to Kaohsiung CGMH with postmenopausal vaginal bleeding in November 2016. Sequential transvaginal ultrasound demonstrated thickened endometrium (1.5 cm thick) and a uterine mass with solid component measuring 5 cm. Histopathology of endometrial curettage showed CCC of the endometrium. The tumor marker carcinoembryonic antigen level was within the normal range, and the carbohydrate antigen 125 (CA-125) level was 25 U/ml. Contrast-enhanced pelvic magnetic resonance imaging confirmed a space-occupying mass (5 x 4.2 x 4.7 cm) over the uterine cavity with invasion to the whole thickness of the myometrial wall and possibly extending to the uterine serosa. An enlarged left external iliac lymph node measuring 1.1 cm was also noted (**Figure 1**).

**Figure 2:**
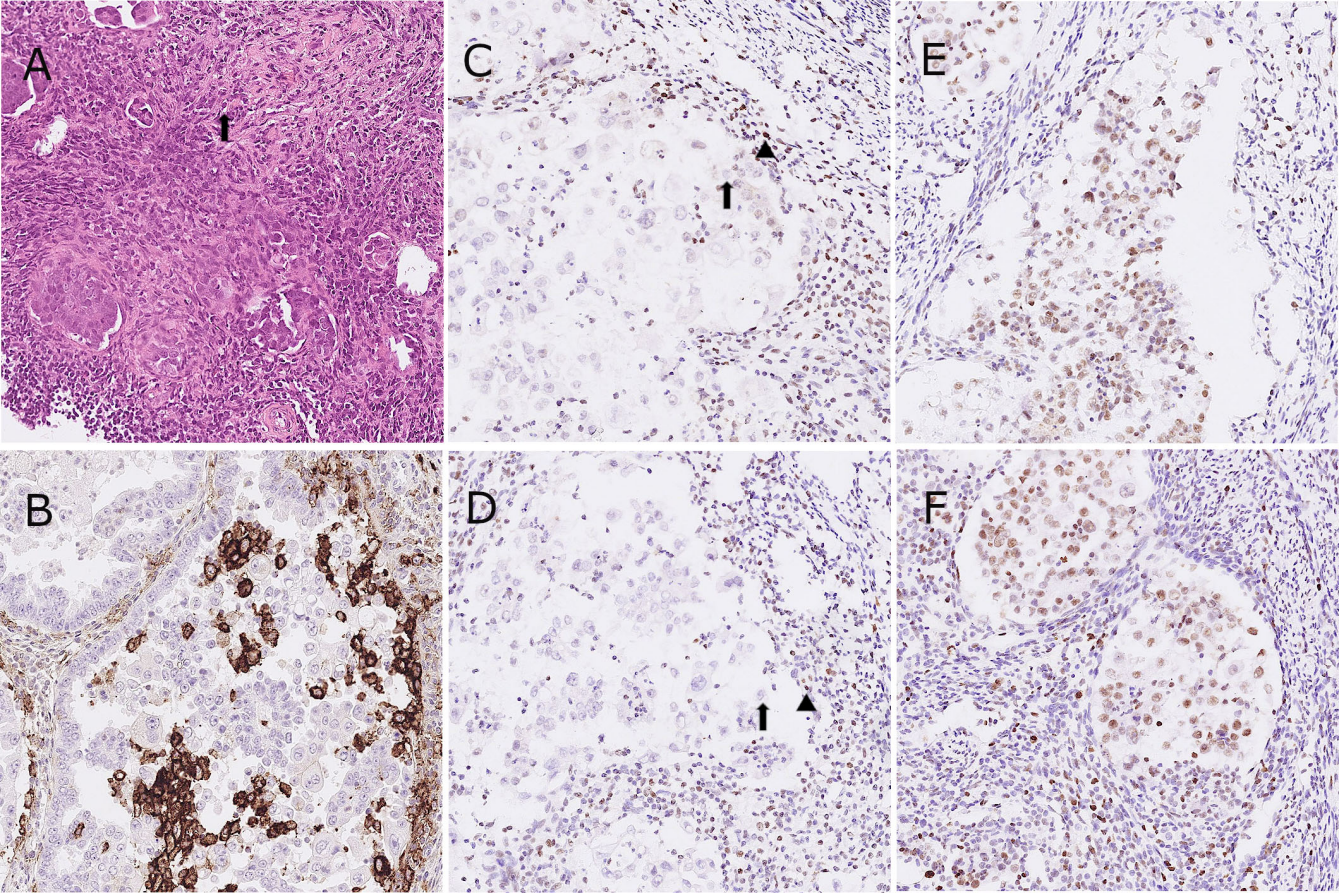
Representative images of hematoxylin and eosin stain **(A)**, tumor cells with a hobnail shape and abundant clear cytoplasm (arrow); immunohistochemistry for PD-L1 **(B)** and four MMR proteins as MLH-1, PMS-2, MSH-2 and MSH-6 in the patient. **(C-F)**, tumor cells with equivocal loss of MLH-1 [arrow, **(C)**], stromal cells for positive control [arrowhead, **(C)**]; tumor cells with complete loss of PMS-2 [arrow, **(D)**], stromal cells for positive control [arrowhead, **(D)**].

The patient underwent complete staging surgery including extended total hysterectomy, bilateral salpingo-oophorectomy, pelvic and para-aortic lymphadenectomy, and omentectomy as primary treatment. The pathological findings revealed endometrial tumor cells with a hobnail shape and abundant clear cytoplasm (**Figure 2A**) positive for HNF-1beta and focally positive for napsin-A in IHC staining, compatible with ECCC with local invasion to the whole layer of the myometrium as well as metastasis to the left ovary and ipsilateral side pelvic lymph nodes, FIGO stage IIIC1. She received 6 cycles of adjuvant chemotherapy with tri-weekly carboplatin and paclitaxel from March 2017 to June 2017. The CA-125 level immediately after the 6th cycle of chemotherapy was 22.4 U/mL. Follow-up computed tomography (CT) immediately after completing adjuvant chemotherapy revealed no definite evidence of recurrent tumors or metastasis in the pelvis.

The CA-125 level increased to 95.0 U/mL 2 months later. Positron emission tomography (PET)/CT showed disease relapse with metastatic lymph nodes in para-aortic, left paravertebral, left supraclavicular and left axillary areas. We further analyzed the endometrial tumor tissue using IHC staining. The IHC staining for PD-L1 showed an expression of 35% according to the combined positive score (CPS) system but less than 1% according to the tumor proportion score (TPS) (**Figure 2B**). In addition, all of the cancer components exhibited an equivocal loss of MLH-1 and complete loss of PMS-2 expression (**Figures 2C–F**).

Based on the defective MMR deficiency in IHC staining, she was started on immune checkpoint inhibitors (ICIs) with tri-weekly injections of pembrolizumab (total 100 mg) in September 2017. Before the treatment, her left neck lymph node was palpable and measured 1.0 by 1.0 cm. After 2 doses of pembrolizumab the lymph node became impalpable, however it emerged and measured more than 0.5 cm following the 4th cycle of pembrolizumab. Therefore, CyberKnife stereotactic body radiation therapy (SBRT) targeting the para-aortic lymph nodes with a total dose of 30 Gy in 3 fractions was administrated in December 2017. After 8 cycles of pembrolizumab, a complete response was achieved according to a follow-up PET/CT scan in March 2018, and the CA-125 level decreased from 111.6 U/mL to 8.2 U/mL. However, she subsequently discontinued immunotherapy due to personal reasons.

**Figure 3:**
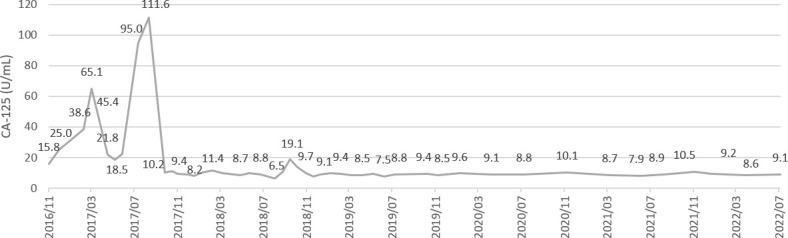
A serial values of tumor marker CA-125 during treatment and follow-up. CA-125 level 111.6 and 19.1 U/ml at 1^st^ and 2^nd^ recurrence respectively.

Disease recurrence was observed again in a PET/CT scan in August 2018, showing progressive changes at the left lower neck and left supraclavicular nodes (**Figure 2**). Hence, we gave her combination therapy with tri-weekly pembrolizumab (total 100 mg) and platinum-based chemotherapy containing doxorubicin (1 mg/kg) + carboplatin (10 mg/kg). Due to intolerance to the side effects of the combination therapy including grade 4 neutropenia, grade 3 nausea/vomiting and grade 2 neurosensory disorder, chemotherapy was discontinued after the 3rd cycle of pembrolizumab. The immunotherapy was well tolerated over 3 months with grade 1 dermatitis and enteritis according to Common Terminology Criteria for Adverse Events (CTCAE) ver. 5.0. Late onset autoimmune thyroiditis occurred 5 months later which was confirmed in a thyroid scan and thyroid echography, and then subsided. She then received 2 years of maintenance therapy with pembrolizumab, and no disease relapse or other significant side effects were noted during treatment. Follow-up PET/CT scans have shown no disease recurrence or metastasis since the discontinuation of immunotherapy, and the CA-125 level has stayed within normal range at less than 10 U/ml. At the time of publication of this report, she has been disease free for 3 years since stopping pembrolizumab treatment (**Figure 3**).

## 3 Diagnostic assessment

### 3.1 Immunohistochemistry (IHC) study

IHC analysis was performed on 3-um sections sliced from tissue array blocks. The primary antibodies used for mismatch repair (MMR) proteins were MLH1 (Genemed Cat# 61-0079-2, RRID : AB_11205257), MSH2 (clone G219-1129, 1:100, ZETA), MSH6 (clone GM024, 1:100, Genemed) and PMS-2 (BD Biosciences Cat# 556415, RRID : AB_396410). The primary ARID1A antibody (Sigma-Aldrich Cat# HPA005456, RRID : AB_1078205) was used at a diluted concentration of 1:2000. Programmed death-ligand 1 (PD-L1) was detected using 22C3 pharmDx Kit (Dako Omnis) anti-PD-L1 (Agilent Cat# GE00621-2, RRID : AB_2833074) mouse monoclonal primary antibody. Non-neoplastic endometrial and stroma cells were used as internal positive controls.

### 3.2 Identification of prognostic biomarkers of immunotherapy using whole-exome sequencing (WES)

DNA was extracted from formalin-fixed paraffin-embedded tissue, and WES of the endometrium tumor and normal endometrial tissue was performed using a commercially available platform (Genomics BioSci & Tech, Taipei, Taiwan). Genomic DNA was isolated from the sections using a Zymo Research Quick-DNA™ FFPE Kit (D3067). A Quant-iT dsDNA HS Assay kit and Qubit Flex instrument were used to measure the concentration of genomic DNA. The integrity of the genomic DNA was assessed using DV200% with a High Sensitivity Large Fragment 50 Kb Analysis Kit and Fragment Analyzer 5200 instrument. The exome hybrid of WES was captured using a SureSelectXT2 target Enrichment System (Agilent Technologies), and then paired-end sequencing was performed using a Novaseq 6000 system (Illumina).

## 4 Discussion

Currently, chemotherapy is the main first-line therapy for recurrent endometrial cancer or advanced metastatic EC. In situations of first-line chemotherapy failure or disease recurrence, second-line chemotherapy has shown disappointing efficacy, with an overall response rate of less than 20% and progression-free survival (PFS) of less than 6 months. In addition, there is also limited data on the efficacy of targeted therapy for recurrent EC with specific molecular subtypes (10). The prevalence of a high tumor mutation burden (TMB) in EC has been estimated to be around 11.2-12.5% (11). Therefore, novel therapies including ICIs have been explored for recurrent or advanced ECCC in some pilot clinical trials (12–14). MMR deficient solid tumors have been shown to have an encouraging response to pembrolizumab (anti-PD-1) with a complete response rate of 21% and partial response rate of 33% in a phase 2 trial (NCT01876511) (12). The KEYNOTE-028 trial further revealed the effectiveness of pembrolizumab for heavily pretreated advanced PD-L1–positive EC, with an objective response rate (ORR) of 13.0% in 23 evaluable patients (13). Moreover, the KEYNOTE-158 trial included solid tumors with high microsatellite instability (MSI) and MMR deficiency, and showed an ORR of 57.1% and a median PFS of 25.7 months in 49 patients with EC. The median duration of response and median OS have not yet been reached (14).

Several combinations of treatment including chemotherapy, radiotherapy and antiangiogenics along with immunotherapy against ovarian CCC and renal CCC have appeared to be beneficial in some clinical trials (15, 16). In our case, ICI in combination with CyberKnife SBRT for metastatic para-aortic lymph nodes were used to improve disease control after the first recurrence. A complete response also was achieved when the treatment strategy was adjusted to ICI combined with chemotherapy after the second recurrence, and the response has been extremely durable even after stopping immunotherapy. Increasing evidence suggests that cytotoxic chemotherapy stimulates immune system activity in patients with cancer (17, 18). Chemotherapy has been shown to change the tumor immune microenvironment by releasing tumor cell-specific antigens and inducing PD-L1 expression in cancer cells (18). The synergistic effects provide an optimal combination strategy as shown in many trials including a wide variety of solid tumor types and grades, so there is a good rationale for their use to improve the anti-tumor immune response (19–21).

Moreover, the combination of radiotherapy and immunostimulants such as ICIs has been reported to boost an abscopal effect (22), which is defined as a metastatic cancer response at a distance from the site of local radiotherapy. In a study on metastatic melanoma, antibodies against cytotoxic T lymphocyte-associated antigen (anti-CTLA4) and anti-PD-L1 reversed T-cell exhaustion and further promoted an abscopal response (23). A possible mechanism is that radiation-induced antigens may activate T cells directly against tumor-specific antigens at both irradiated and non-irradiated tumor sites, which may explain how combination therapy overcomes resistance to ICIs (23). We used this treatment strategy to control distant metastasis of neck lymph nodes with CyberKnife SBRT targeting the para-aortic lymph nodes.

MSI-high or hypermutated phenotypes of EC are categorized as causing a high tumor mutation burden in the TCGA molecular classification, suggesting that immunotherapy has a role in EC treatment (7). In our case, IHC staining exhibited a complete loss of PMS-2 but equivocal loss of MLH-1 expression. Although a higher PD-L1 expression has been reported in MMR-deficient EC compared to MMR-proficient EC, only 3.1% of ECs have been shown to harbor both MSI and PD-1/PD-L1 expressions (24). Although PD-1/PD-L1 expression has been shown to be a prognostic and predictive marker in non-small cell lung cancer, there is little evidence of an association between PD-1/PD-L1 expression and response to ICI in EC (25). In our case, ICIs showed a robust response even though the tumor cells expressed less than 1% PD-L1 in the IHC study, which may echo the discordance of PD-1/PD-L1 expression as a predictive marker in EC. However, the PD-L1 35% expression according to the CPS system in this case was inconsistent to the discordance. Further experimental evidence and clinical data with consideration of the expression of PD-L1 on immune cells are needed to evaluate the role of PD-L1 expression as a predictive marker to outcome of ICI treatment in EC.

**Figure 4:**
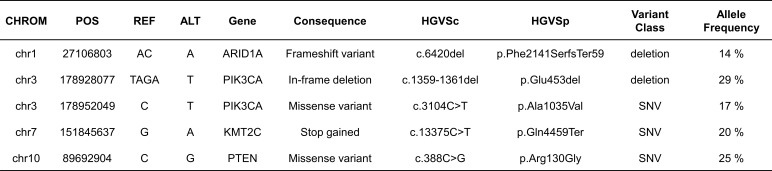
Somatic mutations (SNVs) in WES results. We only reported mutations that are commonly found in ECCC according to the COSMIC database, and only indels, nonsense mutations, and missense mutations that were predicted to be pathogenic.

Furthermore, the WES data in this study revealed that the tumor harbored an intermediate level of TMB with 8.15 mut/Mb and MSI-H. Comprehensive mutation profiles including germline and somatic mutations are shown in **Figure 4**. We only reported mutations that are commonly found in ECCC according to the COSMIC database, and only indels, nonsense mutations (stop-gained), and missense mutations that were predicted to be pathogenic (deleterious). Of the four MMR genes MLH-1, PMS-2, MSH-2 and MSH-6, none had somatic mutations and no pathogenic germline mutation of MLH-1 gene was disclosed. Thus, the equivocal loss of MLH-1 protein expression and complete loss of PMS-2 protein expression may be explained by hypermethylation of the promoter of the MLH-1 gene (26). Frameshift mutations of *ARID1A* were found in the tumor tissues (**Supplementary 1**), which is compatible with most reported *ARID1A* mutations associated with truncated protein production (27). In our case, the complete response after ICI treatment may be due to MMR deficiency and *ARID1A* mutations, suggesting that *ARID1A* status may be a predictive novel biomarker of ICI efficacy for EC (28).

In conclusion, this report describes a rare case of platinum-refractory recurrent ECCC successfully treated with a combination of cytotoxic chemotherapy, radiotherapy and 2 years of maintenance treatment with pembrolizumab. The patient has had an extremely durable response even after stopping immunotherapy for 3 years at the time of publication. The use of ICIs combined with chemotherapy or radiotherapy may have a synergistic effect to activate the immune system. This may contribute to a durable response in MMR deficient EC, and may also be a preferred treatment strategy for recurrence. Furthermore, deficiency of *ARID1A* gene expression may suggest a better efficacy of ICI treatment for EC.

## 5 Patient perspective

This study was conducted in accordance with recognized ethical guidelines and approved by the Institutional Review Board of Chang Gung Memorial Hospital (CGMH), Taiwan. Informed consent was obtained from the patient (number 202100170B0). We confirmed that all experiments were performed in accordance with relevant guidelines and regulations.

## Data availability statement

The original contributions presented in the study are included in the article/**Supplementary Material**. Further inquiries can be directed to the corresponding author/s.

## Ethics statement

The studies involving human participants were reviewed and approved by Institutional Review Board of Chang Gung Memorial Hospital (CGMH), Taiwan (Number 202100170B0). The patients/participants provided their written informed consent to participate in this study.

## Author contributions

CH-K collected data, analyzed data, and wrote the manuscript. CH-W and CT-L were key clinical members for patient care. CH-W designed the study, analyzed data, literature review and edited the manuscript. YC-H and J-L contributed to image data and analysis. All authors contributed to critical revision of the manuscript. All authors approved the prepared manuscript for submission.

## Supplementary material

Supplementary 1 Representative images of immunohistochemical staining for BAF250 protein (encoded by ARID1A gene) in this patient. The tumor cells exhibited weak expression (**A**, arrow) and clonal loss of ARID1A expression (**B**, arrow) as compared to stroma cells.
