# Supplementary figures and images for: Case report: Durable response after pembrolizumab in combination with radiation - induced abscopal effect in platinum - refractory metastatic endometrial clear cell carcinoma
Source: Front Immunol. 2022 Dec 15;13:1079253. doi: 10.3389/fimmu.2022.1079253 (PMC9797856; doi:10.3389/fimmu.2022.1079253)

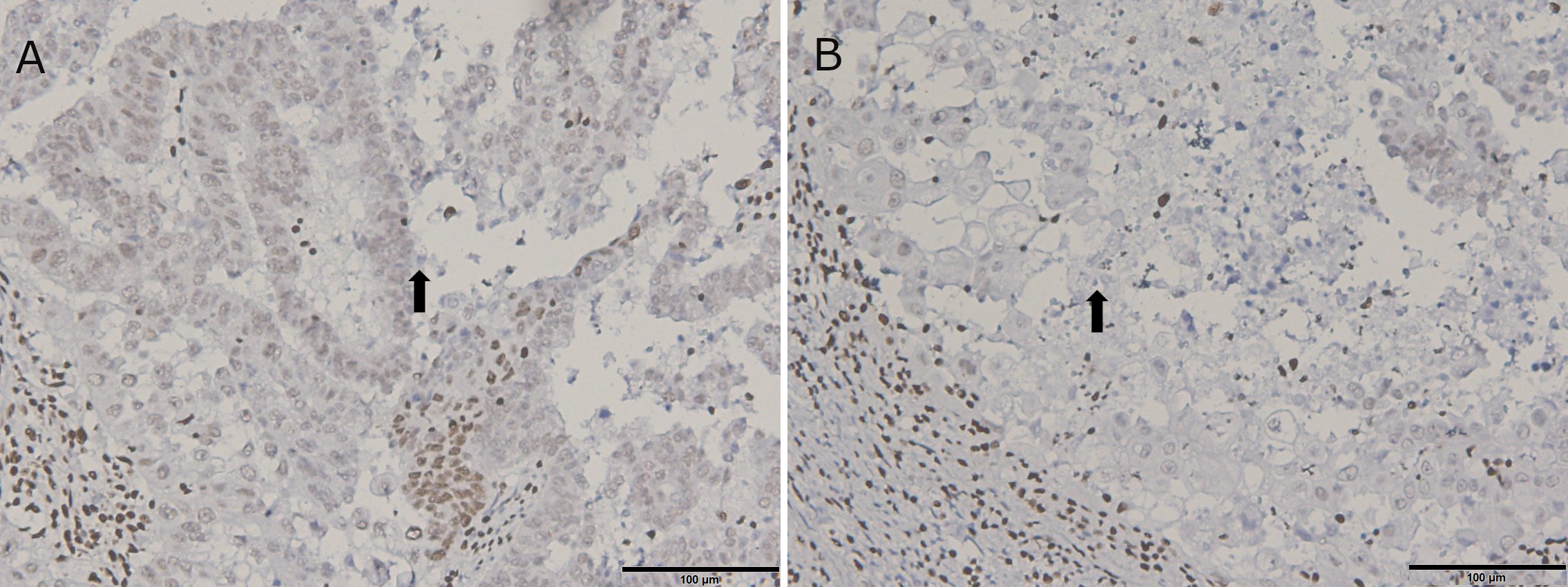

Supplement: Supplementary 1 — Representative images of immunohistochemical staining for BAF250 protein (encoded by ARID1A gene) in this patient. The tumor cells exhibited weak expression (A, arrow) and clonal loss of ARID1A expression (B, arrow) as compared to stroma cells. [file Image_1.tiff]
